# A scalable open-source MATLAB toolbox for reconstruction and analysis of multispectral optoacoustic tomography data

**DOI:** 10.1038/s41598-021-97726-1

**Published:** 2021-10-06

**Authors:** Devin O’Kelly, James Campbell, Jeni L. Gerberich, Paniz Karbasi, Venkat Malladi, Andrew Jamieson, Liqiang Wang, Ralph P. Mason

**Affiliations:** 1 Department of Radiology, University of Texas Southwestern Medical Center, 5323 Harry Hines Blvd., Dallas, TX 75390-9058 USA; 2 BioHPC, University of Texas Southwestern Medical Center, Dallas, TX USA; 3 Lyda Hill Department of Bioinformatics, University of Texas Southwestern Medical Center, Dallas, TX USA

**Keywords:** Cancer imaging, Software

## Abstract

Multispectral photoacoustic tomography enables the resolution of spectral components of a tissue or sample at high spatiotemporal resolution. With the availability of commercial instruments, the acquisition of data using this modality has become consistent and standardized. However, the analysis of such data is often hampered by opaque processing algorithms, which are challenging to verify and validate from a user perspective. Furthermore, such tools are inflexible, often locking users into a restricted set of processing motifs, which may not be able to accommodate the demands of diverse experiments. To address these needs, we have developed a Reconstruction, Analysis, and Filtering Toolbox to support the analysis of photoacoustic imaging data. The toolbox includes several algorithms to improve the overall quantification of photoacoustic imaging, including non-negative constraints and multispectral filters. We demonstrate various use cases, including dynamic imaging challenges and quantification of drug effect, and describe the ability of the toolbox to be parallelized on a high performance computing cluster.

## Introduction

Multispectral optoacoustic tomography (MSOT) is a relatively new imaging modality, which combines optical contrast and ultrasonic resolution in order to provide a highly parametric view of an imaged sample. Due to the high spatiotemporal resolution, MSOT captures vast quantities of information, creating potential challenges: How does one effectively and efficiently analyze such data? Moreover, how does one ensure that the analysis is done in a manner, which is transparent and verifiable, and thus trustworthy, while allowing the flexibility to adjust the manner of processing to accommodate the differential needs of a wide variety of experiments and data acquisition conditions?

To address these issues, we have created an MSOT Reconstruction, Analysis, and Filtering Toolbox (RAFT), enabling users of various technical skill levels to benefit from the rich information present in MSOT data, and to share and compare analyses between sites in a verifiable manner. The package is available as a MATLAB toolbox and a standalone command-line application, with plans to deploy to Docker containers for site- and platform-independence, potential deployment to cloud computing resources, as well as to preserve archival code for execution. The toolbox provides a basis for users to add their own reconstruction and unmixing algorithms, but may also be run in a ‘black-box’ mode, driven only through external configuration files, thus providing greater repeatability. Scalability is addressed by parallelization within MATLAB, allowing computationally intensive reconstructions to be distributed across several computational workers. A Nextflow wrapper around the MATLAB systems allows multiple pipelines to be run on a single computational cluster simultaneously.

## Background

MSOT may be thought of as optically-encoded ultrasound; an object under brief, intense illumination absorbs some of the illuminating energy, converting some of that energy into heat^1,2^. This heat induces transient thermoelastic expansion, creating a pressure wave, which travels outward from the point of absorption. If this absorption and thermal conversion process occurs in a short enough period of time, the pressure wave is temporally confined, creating a compact wavefront, which can be detected using ultrasonic transducers. The reconstruction of the original pressure image then provides a measure of energy deposition by the original illumination, which may be related across multiple illumination wavelengths to yield an overall spectral image. Knowledge of the endmembers present allows the spectral image to be unmixed into its corresponding endmember images.

The promise of MSOT, given recent advancements in laser tuning controls, data acquisition bandwidth, and ultrasound transducer design, is that it can provide optical contrast with ultrasound resolution. Indeed, the modality is highly scalable across various temporal and spatial regimes: It may be used for super-resolution imaging at the scale of tens of nanometers^3–5^, is becoming popular for preclinical and small animal investigations^6–11^ and has been deployed for clinical usage on human subjects^12–18^. The rate of imaging is fundamentally limited by two parameters: The signal to noise ratio achievable by a given photoacoustic imaging technology, and the exposure limits defined by guiding agencies. In practice, the field assumes a maximum energy deposition of 20 mJ/cm^2^ at skin surface, and therefore many low-energy laser shots may be substituted for few high-energy laser shots^19,20^.

Beyond the nature of the acquisition devices themselves, there are numerous methods by which photoacoustic images may be reconstructed; these range from direct inversion algorithms analogous to the ‘delay-and-sum’ approaches of ultrasound^21^, to analytical inversions valid under specialized geometries^22,23^, to model-based approaches analogous to those used in CT and MRI^22,24–29^. Still further methods use the time-reversal symmetry of the governing equations and numerical simulation to determine the original photoacoustic energy distribution^30,31^. These all operate under the governing photoacoustic equations. Under pulsed laser light and the assumption of ideal point transducers, the photoacoustic equations can be written as an optical component (1) and an acoustic component (2)^32^:

1 $$p_{0} \left( {\vec{r},\vec{\lambda }} \right) = p\left( {t = 0, \vec{r},\vec{\lambda }} \right) = H\left( {\vec{r},\vec{\lambda }} \right) = \Gamma \left( {\vec{r}} \right)\phi \left( {\vec{r},\vec{\lambda }} \right)\mu_{a} \left( {\vec{r},\vec{\lambda }} \right) = \Gamma \left( {\vec{r}} \right)\phi \left( {\vec{r},\vec{\lambda }} \right)\mathop \sum \limits_{i = 1}^{{N_{c} }} C_{i} \left( {\vec{r}} \right)\varepsilon_{i} \left( {\vec{\lambda }} \right)$$

2 $$p_{d} \left( {t,\overrightarrow {{r_{d} }} } \right) = \frac{\partial }{\partial t}\left[ {\frac{t}{4\pi }\iint\limits_{{\left| {\vec{r}_{d} - \vec{r}} \right| = \nu_{s} t}} {p_{0} \left( {\vec{r}} \right)d\Omega }} \right]$$

Here, $p$ denotes pressure values, assumed to instantaneously take on the values $p_{0} \left( {\vec{r}} \right)$ at each location $\vec{r}$ throughout the imaging region at time $t = 0$. $H\left( {\vec{r}} \right)$ denotes the heating function, which is dependent on the Gruneisen parameter ${\Gamma }$ describing the efficiency of conversion from light to heat at each point, the light fluence $\phi \left( {\vec{r},\vec{\lambda }} \right)$ defined throughout the imaging region for each illumination wavelength $\lambda$, and the absorption coefficient $\mu_{a} \left( {\vec{r},\vec{\lambda }} \right),$ which describes the conversion of light fluence to absorbed energy at each point and each wavelength. In turn, the absorption coefficient is described by the absorption spectra $\varepsilon_{i} \left( {\vec{\lambda }} \right)$ of all endmembers present at each point, weighted by their concentration at that point $C_{i} \left( {\vec{r}} \right)$ (Eq. 1).

Once light has been absorbed and converted into acoustic waves, these waves spread outward from the point of origin according to the wave equation with wave-fronts traveling at the speed of sound $\nu_{s}$ in the medium, eventually reaching the detection transducers located at $\vec{r}_{d}$, each of which has a solid angle $d{\Omega }$ describing the contribution of each imaged point to the overall measured signal at time $t$ (Eq. 2). This integration over the 2D detection surface is denoted by the double integral in Eq. 2.

Once data have been acquired using transducer geometry $\overrightarrow {{r_{d} }}$ and sampled at $\vec{t}$, the time-dependent photoacoustic data must be reconstructed into the corresponding image. Thorough overviews of the state of the field have been compiled by various groups^1,33–35^. Reconstruction may be accomplished through either direct or inverse methods; the former encompasses such approaches as backprojection algorithms, while the latter encompasses any approach using a model of image formation mapping from $H\left( {\vec{r}} \right)$ to $p_{d} \left( {\vec{t},\overrightarrow {{r_{d} }} } \right)$ and minimizing a cost function. For any given model or reconstruction approach, a variety of parameters and constraints may be applied to the process to enforce certain conditions such as non-negativity, or to regularize the reconstruction process and emphasize certain aspects of the data.

A general representation of the reconstruction process in discrete form is given by the optimization problem

3 $$\vec{I} = \mathop {\text{argmin }}\limits_{{\vec{I}}} \left\| {M\vec{I} - \vec{d}} \right\| + \lambda \left\| {R\left( {\vec{I},\vec{d}} \right)} \right\|$$

where $\vec{I}$ represents the reconstructed image, $\vec{d}$ the acquired data, $M$ the forward model mapping $\vec{I}$ to $\vec{d}$, $R$ a regularization function which is a function of $\vec{I}$ and/or $\vec{d}$, and $\lambda$ a regularization parameter which adjusts the influence of the regularization term in the value of the objective function. Reconstruction may be performed using any norm, though the $L_{2}$ Euclidean norm is most commonly used.

A plethora of pre- and post-processing approaches may be added to the analysis chain; reconstruction may be performed on the individual images, or jointly among several multispectral images simultaneously. Unmixing may be performed prior to, or after, reconstruction, and both reconstruction and unmixing may themselves be combined into a single operation to yield unmixed images directly from multispectral ultrasound data.

It is important that analyses be transparent, verifiable, and validatable. Many approaches may give ‘suitable’ results in a qualitative sense, but applying further quantitative analysis to these less than quantitative intermediate results can lead to spurious analyses and interpretation. It is thus key that assumptions be explicit for any analysis, and that the analytical provenance for a given analyzed datum be recorded. As a salient example, bandpass filtering of the recorded ultrasound signal and deconvolution of the transducer impulse response are common preprocessing steps^36^, but many deconvolution methods require an assumption of white noise, which is violated if bandpass filtering is performed prior to deconvolution.

It is also important that a given analysis be repeatable and consistent across datasets; indeed, if one has a dataset that benefitted from a particular analytical chain, then one would like to easily apply the same processing parameters to other datasets under similar acquisition conditions. However, given the diversity of possible acquisition settings, an analysis must be sufficiently flexible to accommodate the details of each particular dataset, such as the order of wavelengths sampled.

Numerous tools are available to address individual components of this process; K-wave is an excellent toolbox developed over the past several years by Treeby and Cox, largely intended to simulate pressure fields in ultrasound and photoacoustic imaging contexts^37^. Toast++ and NIRFAST are toolboxes to simulate light transport efficiently^38,39^, while MCML addresses light transport from a Monte-Carlo perspective^40^. FIELD-II simulates acoustic sensitivity fields^41,42^. Given the existence of such tools, it is key that they can be effectively leveraged into the new RAFT framework.

Lastly, for any amount of computational power or configurability, it is critical that the use of such tools be straightforward and consistent, and that any changes made to a codebase do not interfere with established analyses or reduce the overall quality of results. It is therefore key that there be means by which the framework is tested and updated automatically, or at least to a level of convenience enabling update and maintenance.

We address several of these problems and believe that this new tool will provide a foundation for the future development of photoacoustic imaging, and that further development of the RAFT will continue to expand its capabilities.

### MSOT-RAFT structure

RAFT operates in a data-driven manner, directed by a configuration file which describes the parameters and methods used to perform a given analysis, and which acts as a record of how a given dataset is processed. Defaults are provided to enable immediate usage, but users may modify these defaults according to their preferences. For settings which are explicitly dependent on metadata, such as the number of samples acquired or the location of transducers, a metadata population step loads such information into a standard form, thereby generalizing the pipeline’s application beyond a specific manufacturer’s technology. Multiple extensions of the processing pipeline to different photoacoustic data are possible through modification with different loaders.

Data frames are loaded using a memory-mapped data interface, allowing large datasets to be handled without loading the entire dataset into memory at one time. This enables the processing to be performed even on workstations with minimal available memory. The action is handled by a loader, which is instantiated using the metadata information associated with the MSOT dataset, giving it a well-defined mapping between a single scalar index and the corresponding data frame. Each raw data frame is associated with acquisition metadata, such as the temperature of the water bath or laser energy, and is output to the next processing step.

**Figure 1:**
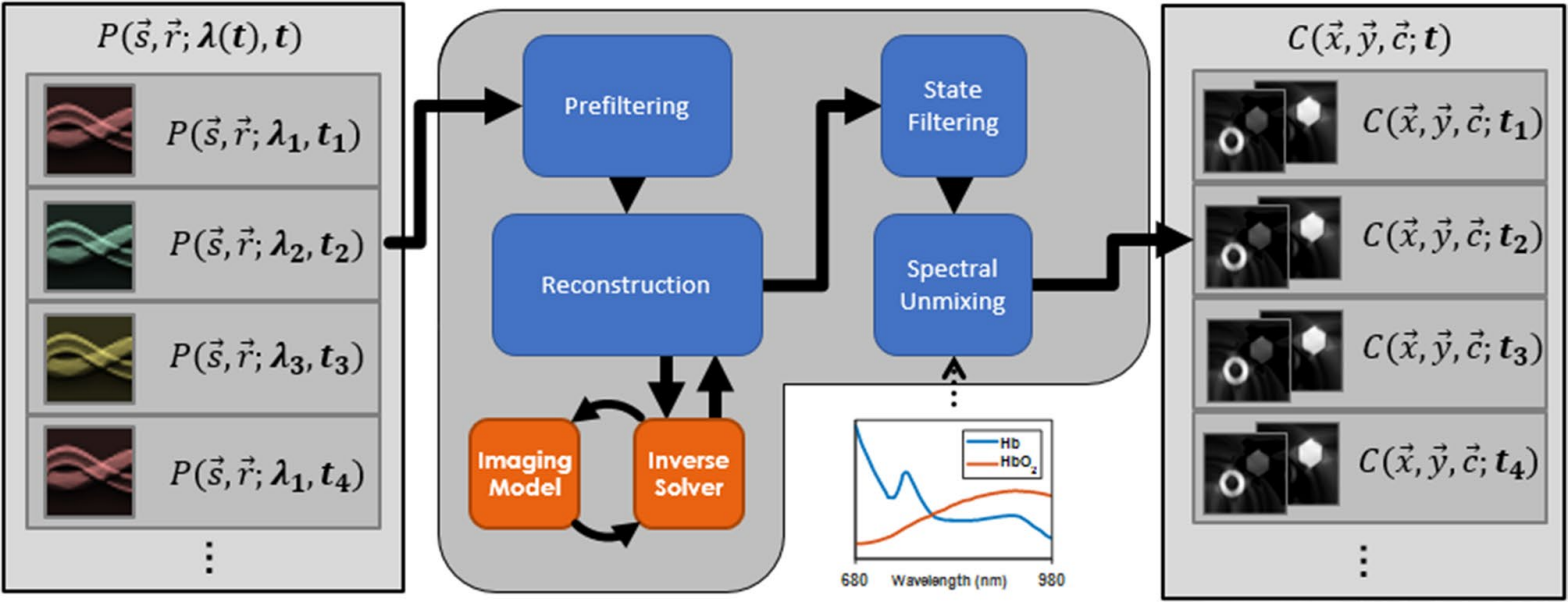
Example pipeline structure. A stream of individual single-wavelength photoacoustic data frames is transformed through a cascade of processing actions to yield a series of spectrally unmixed images at each point in time. The pipeline takes advantage of the known acquisition geometry of the system to perform reconstruction, and user-defined spectral endmembers to perform unmixing. Figure created using a combination of MATLAB and Microsoft PowerPoint.

Processing proceeds via the transformation of data frames, which are arrays of data associated with a particular, well-defined coordinate system. An example is the data frame acquired from a single laser pulse and the resulting acoustic acquisition. Data frames may also have additional, contextual data, such as the time of acquisition or excitation wavelength. Figure 1 illustrates a commonly-used processing topology, with an overall effect of transforming a series of single-wavelength photoacoustic data frames into a series of multi-component image frames.

**Figure 2:**
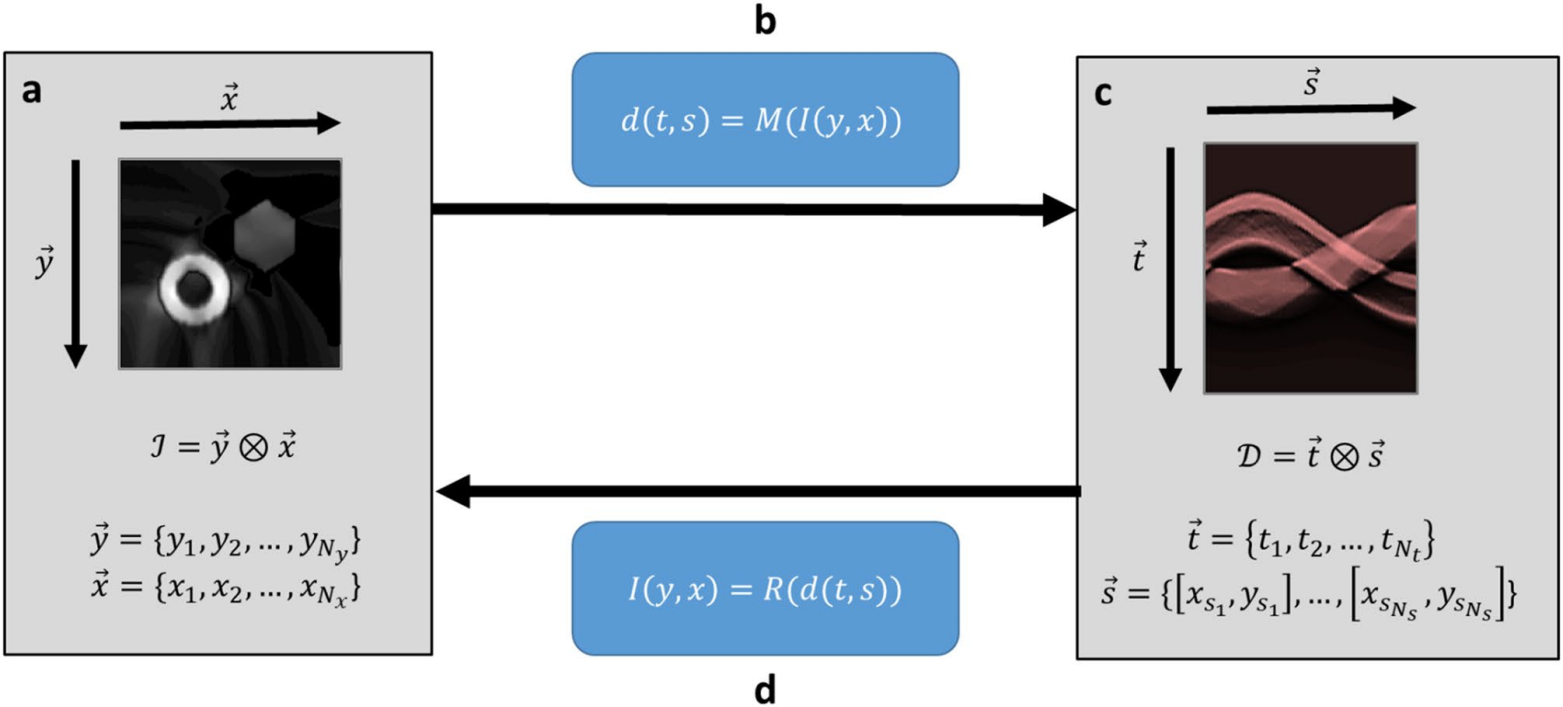
Image-data mappings. An image (**a**) consisting of some explicit values, is organized according to the coordinate system ${\mathcal{I}}$. (**b**) Through the transformation effected by the forward model operator $M$, one determines (**c**) the photoacoustic data in coordinate system ${\mathcal{D}}$. In practice, one acquires the data and (**d**) seeks to reconstruct the corresponding image through the action of some reconstruction operator $R$. Iterative schemes are often favored for the reconstruction process, and so $R$ is implicitly dependent on the operator $M$. Figure created using a combination of MATLAB and Microsoft PowerPoint.

Coordinates explicitly describe the structure of the data contained within data frames. Coordinates may be vectors of scalars or vectors of vectors. An example of the former is the use of two scalar coordinates to describe the X and Y position of a given pixel vertex (Fig. 2a, x and y coordinates), while an example of the latter is an index of transducers, each of which has an associated X,Y position (Fig. 2c, $\vec{\user2{s}}$ coordinate). Transformations between these spaces are effected by the forward model $M$ (Fig. 2b) and the inverse solution operator given in Eq. 3 (Fig. 2d).

Some processing methods require that the procedure maintain memory of its state, *e.g*., in the use of recursive filters or online processing. We therefore implemented the filters using the MATLAB System Object interface, which provides a convenient abstraction to describe such operator mappings, where the transformation may have some time-dependent internal structure. Other processing methods benefit from the assumption that each data frame within a dataset may be treated independently, such as in the case of reconstruction, and the use of an object-oriented design ensures that the processing of such datasets can be effectively parallelized.

### Implementation

The most up-to-date version of the RAFT is available at https://doi.org/10.5281/zenodo.4658279, which may have been updated since the time of writing.

The RAFT is designed in a highly polymorphic manner, allowing different methods to be applied to each step, and for the overall pipeline topology to change. As an example, one can perform multispectral state estimation on the raw data prior to reconstruction, in contrast to the topology shown in Fig. 1, where the multispectral state estimation occurs after reconstruction. For succinctness, we will only be illustrating the pipeline topology shown in Fig. 1.

Following its loading into memory and calibration for laser energy variations, each raw data frame is preconditioned by subtracting the mean of each transducer’s sampled time course, deconvolving the transducer impulse response using Wiener deconvolution^43^, bandpass filtering the signal, correcting for wavelength-dependent water absorption with the assumption of Beer’s law attenuation^44^, and interpolating the data frame into the coordinate system expected by the reconstruction solver.

Reconstruction proceeds by assuming the independence of each data frame, and is thus parallelized across an entire dataset through the use of a number of distributed workers, each performing the same processing on a distinct region of the overall dataset. During initialization, the reconstruction system creates a model operator, which is used during operation to reconstruct each frame into the target image coordinate system. Each frame is then written to disk until processing completes.

Spectral unmixing is accomplished by providing a stream of single-wavelength data frames to a multispectral state filter, which estimates the true multispectral image at each point in time^45^. This multispectral image estimate is then provided to another solver system which inverts the mixing model, derived from the known wavelength space and the assumed endmembers present.

To readily accommodate future methods, we established a consistent input configuration for processing steps. Reconstruction systems are constructed by pairing an inverse solver to a forward model: The forward model represents the mapping from some input space (here, an image) to some output space (here, a frame of photoacoustic data), while the inverse solver takes the model as an argument and attempts to reduce some objective function subject to some input arguments. Models have a standardized initialization signature, requiring an input coordinate system, an output coordinate system, and a set of model-specific parameters, which, in the case of MSOT, necessarily includes the speed of sound of the medium.

### Pipeline parallelization

MSOT data processing is computationally intensive, and the associated large problem sizes incur substantial processing time. In biological research, where cohorts of animals may be assessed multiple times over the course of a study, this processing time can result in prohibitively long experimental iterations, hindering effective development of methods. If there is no co-dependence among individual acquisitions, it is possible to parallelize computationally intensive steps, and particularly desirable to do so when such steps require a long time to process^46,47^. We therefore implemented the toolbox using MATLAB’s object-oriented functionalities to enable one processing system to be copied among an arbitrary number of parallel workers, and to demonstrate the benefits of parallelization in the case of reconstruction. When many datasets are to be processed using the same configuration, it is desirable to parallelize the processing at the scale of datasets. To this end, we implemented a Nextflow wrapper around the toolbox to illustrate a possible scenario of deploying the toolbox on a computational cluster. With a cluster scheduler, an arbitrary number of datasets can be processed simultaneously, enabling substantial horizontal scalability.

### Comparison of processing methods

There is a practically infinite number of combinations and permutations of different reconstruction methods, preconditioning steps, solver settings, cost functions, and analyses, resulting in a highly complex optimization problem. By providing means to generate arbitrary amounts of test data in a parametric fashion, and by describing processing pipelines using well-defined recipe files, we enable optimization of the complex parametric landscape describing all possible processing pipelines. We illustrate the use of the pipeline to assess the effects of different processing approaches in providing an end analysis.

## Methods

Results were processed using MATLAB 2017b (MathWorks, Natick, MA), though we include continuous integration testing intended to provide consistency between versions. All figures were created using a combination of MATLAB and Microsoft PowerPoint.

All animal work was conducted under animal protocol (APN #2018-102344-C) approved by the UT Southwestern Institutional Animal Care and Use Committee. All animal work was conducted in accordance with the UT Southwestern Institutional Animal Care and Use Committee guidelines as well as all superseding federal guidelines and in conformance with ARRIVE guidelines**.**

We verified the ability of the pipeline to perform reconstructions through the use of several testing schemes. First, we implemented analytical data generation, both single-wavelength and multispectral, in order to test the numerical properties of models and reconstructions. Data were generated consisting of random paraboloid absorbers across a field of view, along with the corresponding photoacoustic data, for a variety of pixel resolutions. For each model, 50 random images were generated with random numbers of sources, random sizes, and random locations at each of the chosen resolutions, and the correspondence of each model’s forward data to the analytical data was quantified. The assumption of point detectors allows one to find the analytical signal expected at a given sampling location, and the assumption of linearity allows one to calculate the total signal from several non-overlapping sources. This provides validation of imaging models, by comparing the known image of sources against the model output. We use this process to demonstrate the potential use of the RAFT as a process optimization tool, informing selections of methods and parameters under different use-cases.

To test the performance of the different models and solvers at different imaging resolutions, we generated numeric data of paraboloid absorbers. The span of each paraboloid was constrained to lie in the field of view of the reconstructed image. Given a known set of paraboloid parameters, it is possible to construct the corresponding projection of the paraboloids onto the image space and the data space. Given the known source image, we compared the output of each model to the ground truth data. To compare solvers, given the output data, we compared the reconstructed image against the known ground truth image, creating an error image (Eq. 4). We quantified the mean bias (Eq. 5), average L_1_ (Eq. 6) and L_2_ norms (Eq. 7), scaled by the number of pixels in an image frame or reconstruction:

4 $$e\left( {x,y} \right) = I_{known} \left( {x,y} \right) - I_{recon} \left( {x,y} \right)$$

5 $$bias = \frac{{\mathop \sum \nolimits_{{y \in {\mathcal{Y}}}} \mathop \sum \nolimits_{{x \in {\mathcal{X}}}} e\left( {x,y} \right)}}{{N_{x} N_{y} }}$$

6 $$\left| {\left| {\vec{e}} \right|} \right|_{{L_{1} }} = \frac{{\mathop \sum \nolimits_{{y \in {\mathcal{Y}}}} \mathop \sum \nolimits_{{x \in {\mathcal{X}}}} \left| {e\left( {x,y} \right)} \right|}}{{N_{x} N_{y} }}$$

7 $$\left| {\left| {\vec{e}} \right|} \right|_{{L_{2} }} = \frac{{\sqrt {\mathop \sum \nolimits_{{y \in {\mathcal{Y}}}} \mathop \sum \nolimits_{{x \in {\mathcal{X}}}} e\left( {x,y} \right)^{2} } }}{{N_{x} N_{y} }}$$

The bias of the reconstruction signifies the tendency of the reconstruction process to over- or under-estimate the pixel values of the reconstructed image—bias values close to 0 indicate that the reconstructed values are on average equal to the true values. The L_1_ and L_2_ norms reflect the error in the reconstruction—lower values of each indicate that the solution converges to a more precise estimate of the true image values.

We additionally quantified the Structural Similarity Index (SSIM)^48^, a measure of image similarity, as a scale-invariant figure of merit:

8 $$SSIM_{{x\hat{x}}} = \frac{{\left( {2\mu_{x} \mu_{{\hat{x}}} + C_{1} } \right)\left( {2\sigma_{{x\hat{x}}} + C_{2} } \right)}}{{\left( {\mu_{x}^{2} + \mu_{{\hat{x}}}^{2} + C_{1} } \right)\left( {\sigma_{x}^{2} + \sigma_{{\hat{x}}}^{2} + C_{2} } \right)}}$$

The average norms and the SSIM were calculated for the error images for each generated phantom image, and this process was repeated N = 50 times at each resolution tested ($N_{x} = N_{y} \in \left[ {30, 50, 100, 150, 200, 250, 300, 350, 400} \right]$). For the solver comparisons, we additionally calculated the number of negative pixels in each image as well as the relative residual at convergence for each method, with the relative residual given by:

9 $$r_{rel} = \frac{{\left\| {M\vec{I} - \vec{d}} \right\|}}{{\left\| {\vec{d}} \right\|}}$$

To demonstrate application to biological data, we analyzed two preclinical imaging scenarios: The first (Dataset A) was a dynamic observation of a gas challenge followed by administration of a vascular disrupting agent (VDA). A male NOD-SCID mouse (Envigo) with a human PC3 prostate tumor xenograft implanted subcutaneously in the right aspect of the back was subjected to a gas breathing challenge, while continuously anesthetized with 2% isoflurane. Prior to imaging, the animal was shaved and depilated around the imaging region to avoid optical or acoustic interference from fur. Gas flow was maintained at 2L/min throughout the imaging session and mouse was allowed to equilibrate in the imaging chamber for at least 10 min before measurements commenced. Initial air breathing was switched to oxygen at 8 min, back to air at 16 min, and again to oxygen at 24 min. At 34 min, the animal was given an intraperitoneal injection of 120 mg/kg combretastatin A-4 phosphate (CA4P) in situ^49–51^, and observed for a further 60 min. Imaging proceeded by sampling the wavelengths [715, 730, 760, 800, 830, 850] nm, with each wavelength oversampled 6 times, but not averaged. Overall, 52,091 frames of data were acquired.

The second scenario (Dataset B) examined a single gas challenge, wherein a female nude mouse (Envigo) was implanted with a human MDA-MB-231 breast tumor xenograft in the right dorsal aspect of the lower mammary fat pad. The gas challenge consisted of 11 min breathing air, 8 min breathing oxygen, followed by 10 min breathing air. Imaging proceeded by sampling the wavelengths [715, 730, 760, 800, 830, 850] nm, with each wavelength oversampled 2 times but not averaged. Overall, 11,184 frames were acquired.

We tested the scalability of the pipeline by performing reconstruction of two experimentally-acquired datasets of 52,091 frames (Dataset A) and 989 frames (Dataset C, acquired during a tissue-mimicking phantom experiment), using two distinct indirect methods and one direct method. Reconstruction was performed using either the Universal Backprojection (BP) algorithm^21^, chosen for its intrinsic universality, or the closely related direct interpolated model matrix inversion^28^ (dIMMI) or curve-driven model matrix inversion^26^ (CDMMI) models. The two models differ in how they discretize the photoacoustic imaging equations. The dIMMI model uses a piecewise-linear discretization with a defined number of points sampled along the wavefront, and uses bilinear interpolation to assign weights to nearby pixels. CDMMI, in contrast, assumes a spherical wavefront and exactly calculates the arc-length of the wavefront within each pixel. The solvers used for reconstruction were either MATLAB’s built-in LSQR function with default parameters, or the non-negative accelerated projected conjugate gradient (nnAPCG) method^52^. The total processing time for reconstructing all frames of Dataset A was determined using $N = \left[ {4,8,12,16,20,24} \right]$ distributed workers, while $N = \left[ {1,2, \ldots ,24} \right]$ distributed workers were applied to Dataset C.

We used the RAFT to execute two distinct processing pathways on each biological dataset, using the package’s default parameter settings except where otherwise specified: The first, so-called ‘unconstrained’ method, used the dIMMI model and an LSQR solution of the imaging equations, and a sliding-window multispectral state estimation filter to produce a complete multispectral image at each point in time.

Unmixing was then performed using the pseudoinverse of the mixing equations:

10 $$\begin{aligned} I\left( {\vec{r},\vec{\lambda }} \right) & = \left( {\begin{array}{*{20}c} {\vec{I}_{{\lambda_{1} }} \left( {\vec{r}} \right)} \\ \vdots \\ {\vec{I}_{{\lambda_{{N_{\lambda } }} }} \left( {\vec{r}} \right)} \\ \end{array} } \right) = \left( {\begin{array}{*{20}c} {I_{{\lambda_{1} ,r_{1} }} } & \cdots & {I_{{\lambda_{1} ,r_{{N_{r} }} }} } \\ \vdots & \ddots & \vdots \\ {I_{{\lambda_{{N_{\lambda } }} ,r_{1} }} } & \cdots & {I_{{\lambda_{{N_{\lambda } }} ,r_{{N_{r} }} }} } \\ \end{array} } \right) \approx \mu_{a} \left( {\vec{r},\vec{\lambda }} \right) = \mathop \sum \limits_{i = 1}^{{N_{c} }} C_{i} \left( {\vec{r}} \right)\varepsilon_{i} \left( {\vec{\lambda }} \right) = {\text{\rm E}}\vec{C} \\ & = \left( {\begin{array}{*{20}c} {\varepsilon_{{\lambda_{1} ,C_{1} }} } & \cdots & {\varepsilon_{{\lambda_{1} ,C_{{N_{c} }} }} } \\ \vdots & \ddots & \vdots \\ {\varepsilon_{{\lambda_{{N_{\lambda } }} ,C_{1} }} } & \cdots & {\varepsilon_{{\lambda_{{N_{\lambda } }} ,C_{{N_{c} }} }} } \\ \end{array} } \right)\left( {\begin{array}{*{20}c} {c_{{C_{1} ,r_{1} }} } & \cdots & {c_{{C_{1} ,r_{{N_{r} }} }} } \\ \vdots & \ddots & \vdots \\ {c_{{C_{{N_{c} }} ,r_{1} }} } & \cdots & {c_{{C_{{N_{c} }} ,r_{{N_{r} }} }} } \\ \end{array} } \right) \\ \end{aligned}$$

11 $$\hat{C} \left( {\vec{r},\vec{c}} \right) \approx {\text{\rm E}}^{ + } \vec{I}\left( {\vec{r},\vec{\lambda }} \right)$$

Here, $\vec{I}_{{\lambda_{j} }} \left( {\vec{r}} \right)$ represents the spectral image at each wavelength $\lambda_{j}$ and each pixel location $\vec{r}$. Variations in fluence are assumed to be negligible, and so the multispectral image is assumed to be approximately equivalent to the actual absorption image $\mu_{a}$. The mixing matrix ${\text{\rm E}}$ is constructed by taking the molar absorption coefficients of each endmember $C_{i}$ at each wavelength $\lambda_{j}$. The unmixed concentration image is thus derived by multiplying $I(\vec{r},\vec{\lambda }$) on the left by the pseudoinverse ${\text{\rm E}}^{ + }$, itself calculated by using MATLAB’s *pinv* function. In this work, the endmembers were assumed to be only oxyhemoglobin and deoxyhemoglobin, with values derived from the literature^53^.

The second, so-called “constrained” approach, used the CDMMI method and an nnAPCG solution of the imaging equations to provide a non-negativity constraint, and an $\alpha \beta$ Kalata filter^45^ for the multispectral state estimation at each point in time to provide a kinematic constraint on the time-evolution of the signal. Unmixing was then performed using the nnAPCG solver applied to the mixing equations. The second imaging scenario was additionally processed using an second constrained approach, using an $\alpha$ Kalata filter. The $\alpha$ filter is very effective at reducing noise over time, but is unable to reliably track rapid dynamic changes in the underlying signal, causing a lagged response. The $\alpha \beta$ filter, by contrast, has lower noise-suppression properties but is much more able to follow signal dynamics. This is in essence due to the fact that the $\alpha$ filter assumes the underlying signal is static.

All approaches were preconditioned by first subtracting the mean from each transducer’s pressure data, Wiener deconvolution of the impulse response function, bandpass filtering between 50 kHz and 7 MHz, and correction by the water attenuation coefficient for a given frame’s acquisition wavelength, assuming a general path length of 3 cm. All images were reconstructed at 200 × 200 resolution and a 2.5 cm field of view unless otherwise specified. The effects of the combination of these steps can be seen in Supplementary Fig. 1.

We assessed the variation in quantitation by several approaches. For Dataset A, we quantified the indicator function $H^{ - } \left( x \right)$ of negative pixels throughout, *i.e*., the number of times each pixel in the reconstructed images attained a negative value throughout the imaging session. The occurrence of negative pixels creates interpretation difficulties, and it is desirable to have few or no negative pixels in a dataset. The unmixed values of hemoglobin ([Hb]^MSOT^), oxyhemoglobin ([HbO_2_]^MSOT^), total hemoglobin ([Hb_Tot_]^MSOT^), and oxygen saturation (SO_2_^MSOT^) were compared between the two methods using a randomly-sampled binned Bland–Altman analysis with 100 × 100 bins, randomly sampling 10% of all pixels throughout the image time course. In Dataset A, the significance of the difference in each unmixed parameter before and after drug was quantified, using a two-population T-test and N = 100 frames between [19900:20000] frames and [51900:52000], corresponding to 10 s immediately before administration of the drug and 10 s immediately before the end of the imaging session. The T-score itself was used as the figure of merit. We additionally quantified the relative effect of the CA4P response by using the initial gas challenge as a measure of scale, relating the Cohen’s D of the CA4P response to the Cohen’s D of the transition from oxygen-air at 16 min. This was done based on the presumption that the initial gas challenge provides indications of the patency of vasculature, which should be related to the response to CA4P. Details of this analysis are presented in Supplementary Information.

The MDA-MB-231 gas challenge was assumed to have a rectangular input pO_2_ waveform. We quantified the centered correlation of each pixel in each channel for each method against this known input function, and calculated the mean and variance of the correlation within tumor, spine, and background ROIs, as well as an ROI covering the whole animal cross-section. Statistical significance of differences between each method’s correlation coefficient was quantified using Fisher’s Z transformation on the raw correlation coefficients and a two-population Z-test. Significance was set at $\alpha = 0.01$, while strong significance was set at $\alpha = 0.0001$.

We additionally tested the ability of each of the processing approaches (Unconstrained, Constrained + $\alpha \beta$, Constrained + $\alpha$) to provide results which were amenable to further analysis, in this case fitting a 7-parameter exponential model (Supplementary Methods) to the [HbO_2_]^MSOT^ time course in each pixel for each method.

Computation was performed using the UT Southwestern BioHPC computational resource; timing was calculated using computational nodes with between 256 and 384 GB of RAM and 24 parallel workers.

## Results

**Figure 3:**
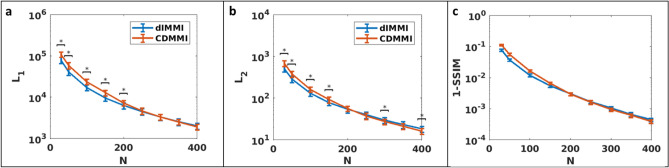
Comparison of dIMMI and CDMMI models. The dIMMI model has statistically superior performance in accurately modelling the photoacoustic imaging process for low image resolutions (**a**, **b**) under both the L_1_ and L_2_ norms. At high resolutions, CDMMI trended towards superior performance over dIMMI, though does not consistently achieve statistical separation. (**c**) Both dIMMI and CDMMI have improved SSIM scores as a function of pixel resolution; CDMMI achieves slightly better performance at high resolutions. Figure created using a combination of MATLAB and Microsoft PowerPoint.

When comparing CDMMI to dIMMI in terms of accurate prediction of model output data, both methods performed with statistical equivalence at high resolutions (Fig. 3). At lower resolutions, there was a notable increase in the modelling error of the CDMMI method. This may be attributed to the problem of aliasing; the dIMMI method allocates additional sampling points along the integral curve during model calculation, acting as an anti-aliasing filter, which reduces the incidence of high-frequency artifacts exacerbated by the differentiation step. However, the CDMMI model seems to indicate a trend towards statistically improved performance at higher resolutions.

**Figure 4:**
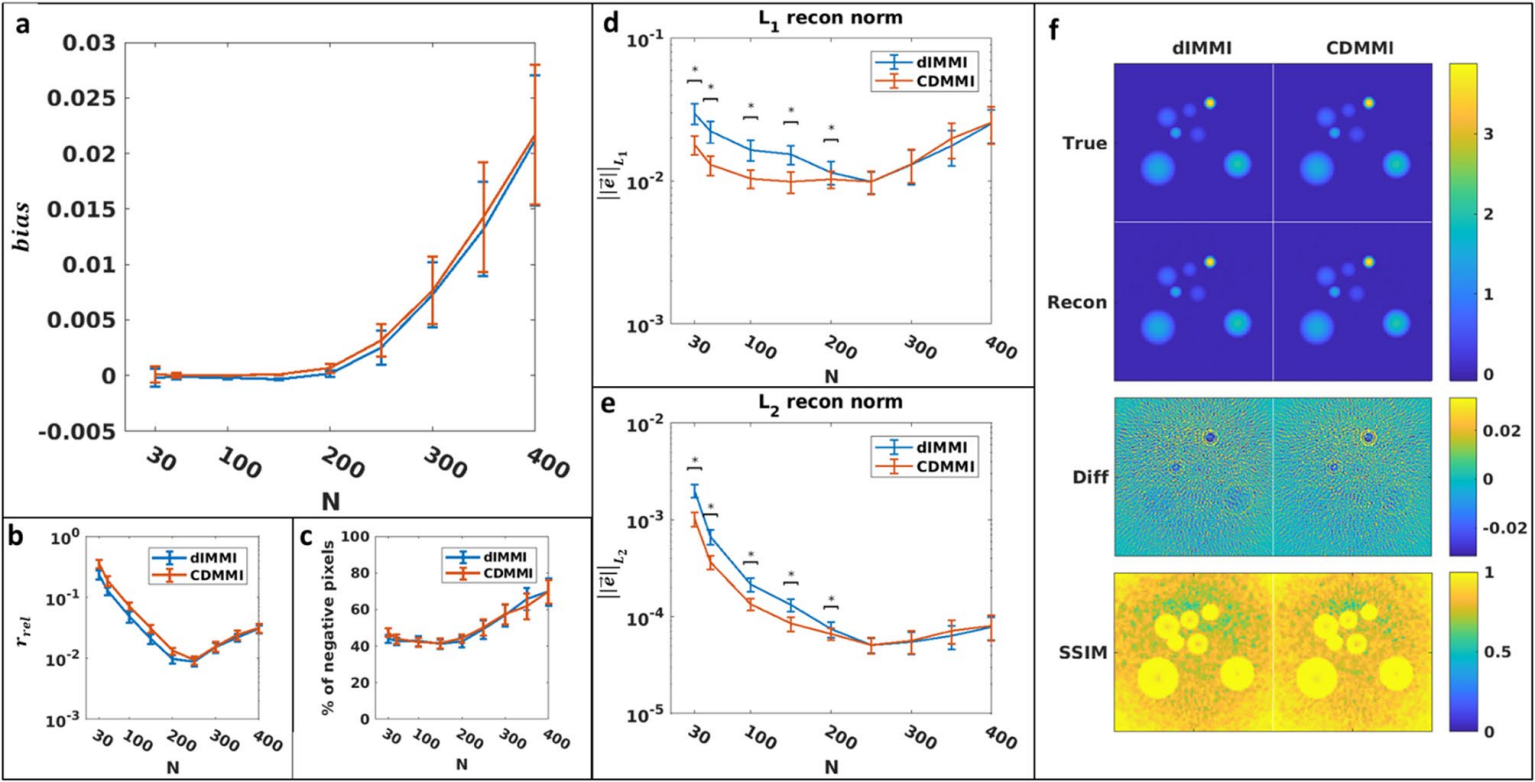
Comparison of dIMMI- and CDMMI-based reconstructions using the LSQR reconstruction algorithm. (**a**) Both models have a tendency towards increasing absolute error as a function of image size, though (**b**) the relative residual for CDMMI is higher than dIMMI for low image resolutions, converging at higher resolutions. (**c**) Both models incur similar numbers of negative pixels in their reconstructions. These trends are reversed, however, when considering the reconstruction L_1_ norm (**d**) and L_2_ norm (**e**) for each model, with CDMMI outperforming dIMMI when reconstructing at low resolutions; again, both methods perform similarly at high resolutions. (**f**) depicts the ground truth, reconstructed, difference, and SSIM images for each of the methods. Both methods appear to perform similarly well, and the introduction of reconstruction artifacts as seen in the SSIM images affects both to comparable degrees. Figure created using a combination of MATLAB and Microsoft PowerPoint.

**Figure 5:**
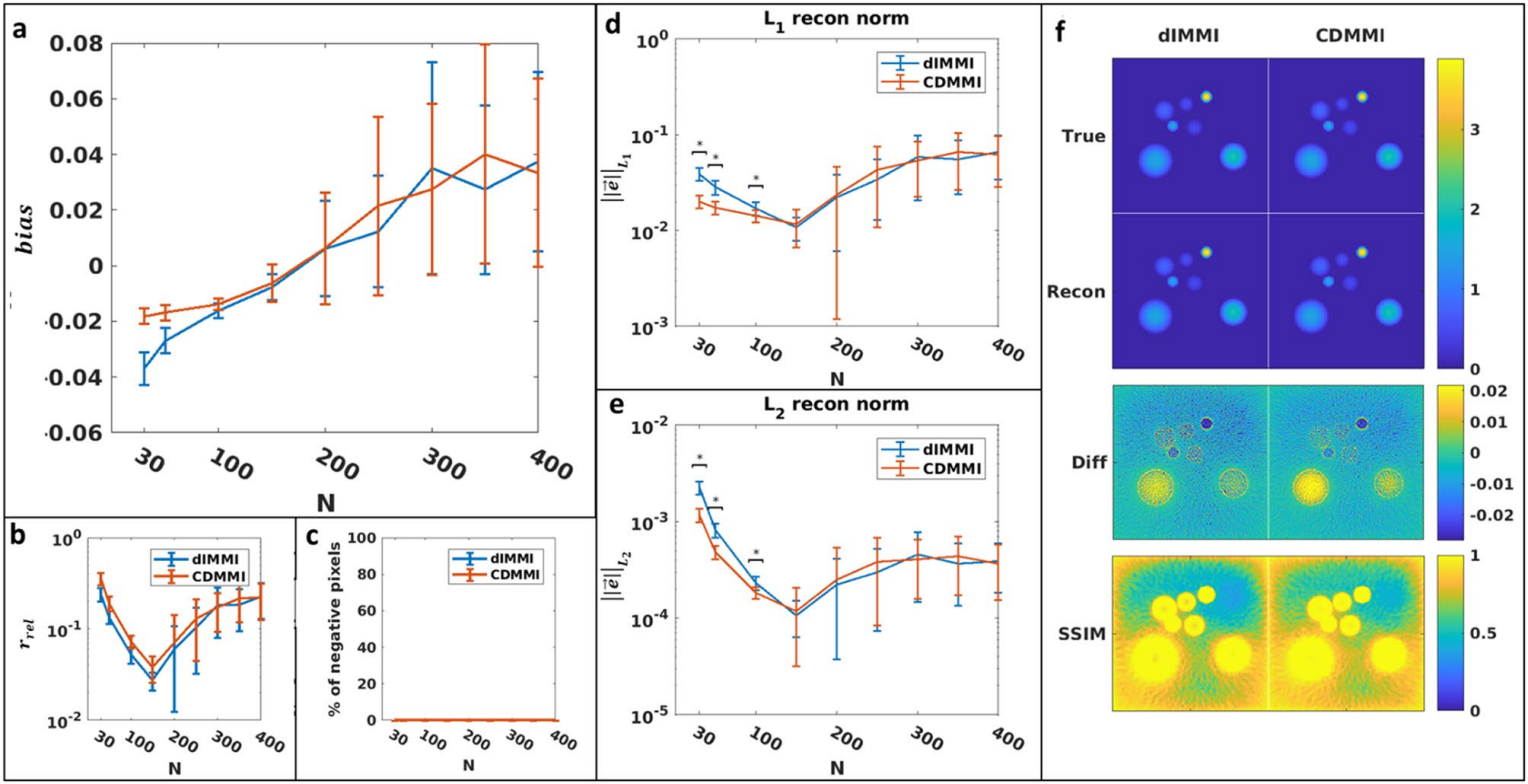
Comparison of dIMMI- and CDMMI-based reconstructions using the nnAPCG reconstruction algorithm. (**a**) Both models tend towards increasing absolute error as a function of image size though (**b**) the relative residual for CDMMI is higher than dIMMI for low image resolutions, converging at higher resolutions. (**c**) Neither model incurs negative pixels due to the non-negative constraint. When considering the reconstruction L_1_ norm (**d**) and L_2_ norm (**e**) for each model, CDMMI results in a lower norm at low resolutions; again, both methods perform similarly at high resolutions. (**f**) depicts the ground truth, reconstructed, difference, and SSIM images for each of the methods. Both models underestimate the true image intensity for high-intensity objects when reconstructed using nnAPCG, causing mismatches towards the center of each object. Figure created using a combination of MATLAB and Microsoft PowerPoint.

Reconstruction performance contrasts the modelling results (Figs. 4 and 5); when images were reconstructed using the LSQR method (Fig. 4) and considering the L_1_ and L_2_ norms (Fig. 5d, e), the CDMMI method had statistically superior performance over dIMMI (N < 250), though at higher resolutions the difference was negligible. In contrast, when considering the relative residual (Fig. 4b), dIMMI provided superior reconstruction performance. Similar results were seen for the nnAPCG method (Fig. 5), though we note the minimum relative residual, L_1_ norm, and L_2_ norm were achieved at a lower resolution (N = 150). This discrepancy between the resolutions of minimum relative residual or norm potentially indicates a need for additional iterations at higher resolutions for the nnAPCG method. We note, however, that the presence of a minimum indicates that the RAFT itself may be optimized through the use of randomly-generated data to provide optimal reconstruction settings.

The choice of distinct reconstruction methods has a salient impact on the quality of the reconstruction, as well as the sensitivity of the method to solver parameters. The solution process in MSOT does not necessarily converge after infinite iterations due to ill conditioning of singular values, and so must be halted at an early stage, or a truncated singular value decomposition (T-SVD) be used as a regularization process. This explains the higher variation in the various error metrics shown in Figs. 4 and 5—LSQR internally uses a more numerically stable algorithm and converges more efficiently than the fundamentally nonlinear nnAPCG method. Together with convergence, a similar question applies to analytical quality; although the absolute error of a given reconstruction may be low in a numerical or simulated environment, artifacts may appear in real data due to nonlinearities and modelling inaccuracies, which are not fully captured by the forward models. LSQR, despite its favorable numerical properties, provides no guarantees of converging to a sensible value, and due to the non-local nature of the photoacoustic imaging equations, errors or nonphysical values in one pixel will affect the reliability of values in other pixels. Due to this combination of factors, we recommend the use of the nnAPCG method when possible, as it provides an inherently constrained reconstruction suitable for use in further analyses, particularly regarding spectral unmixing. Indeed, the use of a non-negative analysis constrains the possible SO_2_^MSOT^ values to the range of [0,1] instead of [$- \infty ,\infty$], which may result from various combinations of positive and negative values of oxy- and deoxy-hemoglobin.

**Figure 6:**
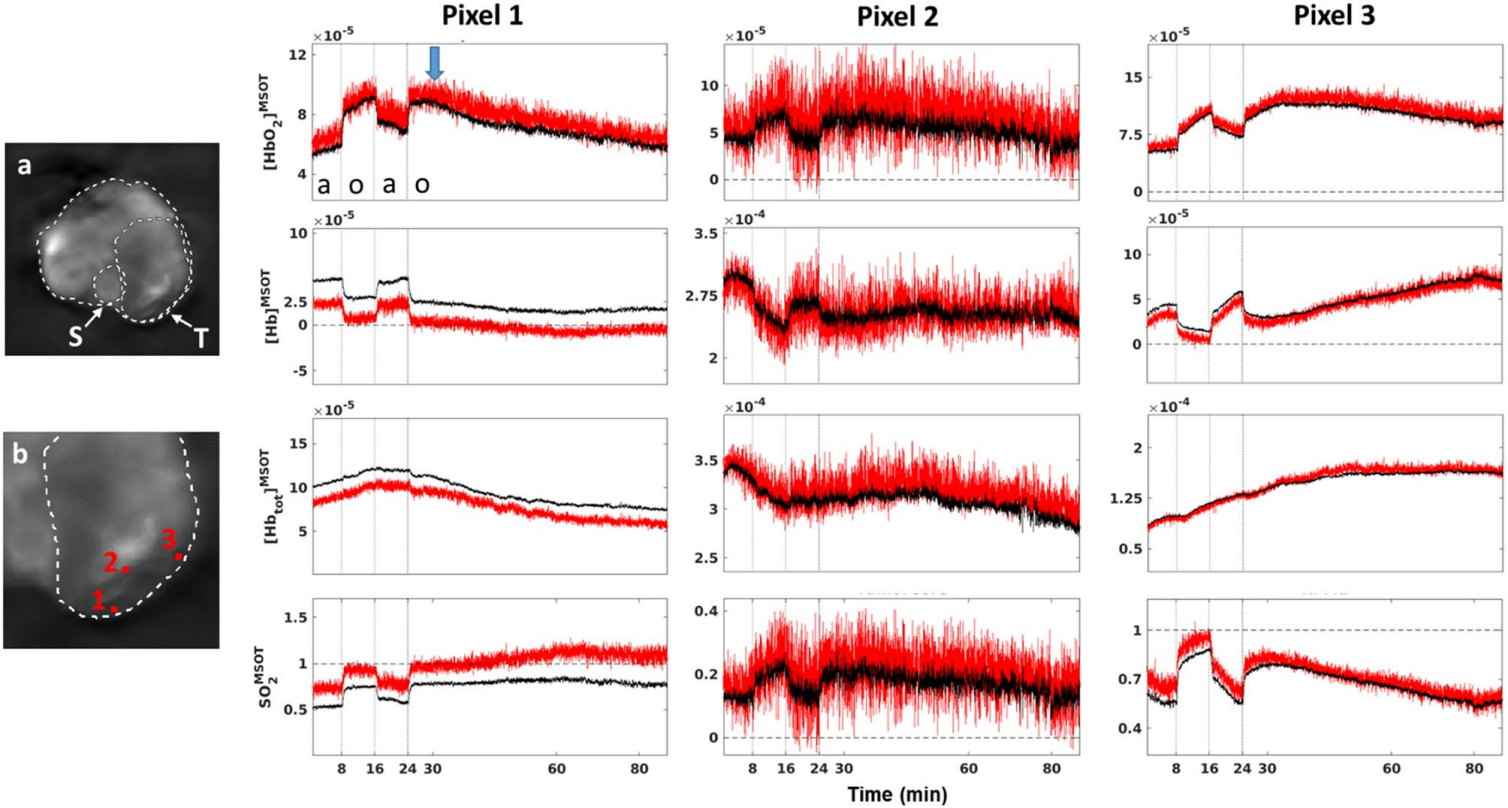
Comparison of different processing approaches implemented using the MSOT-RAFT for analysis of Dataset A. (**a**) Mean cross-sectional [Hb_tot_]^MSOT^ image. S: Spine, T: Tumor. (**b**) Expanded view of tumor periphery, with pixels of interest highlighted; relative size of highlighted squares is larger than a single pixel to improve visibility. The unconstrained approach (red) is substantially noisier and regularly incurs non-physical negative values in the low-signal outer tumor (Pixel 1, [Hb]^MSOT^), which result in spurious values exceeding 1 for downstream SO_2_^MSOT^ calculation (Pixel 1, SO_2_^MSOT^). Poor SNR as seen in Pixel 2 is managed with an $\alpha \beta$ filter (black), making the transitions between gases much more conspicuous. The $\alpha \beta$ filter preserves the dynamical structure of each pixel as well, as seen in the transitions of Pixel 3. Figure created using a combination of MATLAB and Microsoft PowerPoint.

Figure 6 illustrates differences in reconstructed performance for three individual pixels in distinct regions of a tumor in a mouse, each showing specific cases within a single study where the constrained approach provides benefits. Though the shapes of the time courses of each parameter within each pixel are consistent between methods, it is clear that the constrained method produces less noisy data. This is due in large part to the use of the $\alpha \beta$ multispectral Kalata filter, which reduces inter-frame noise^45^. The unconstrained approach (red) also demonstrates the risks of using inappropriate analyses when processing data. The [Hb]^MSOT^ values in the tumor pixel were negative for a large portion of the experiment (Fig. 6, Pixel 1), which resulted in [SO_2_]^MSOT^ values exceeding 1. Such pixels can corrupt both spatial and temporal averages, but are not readily compensated; at the same time, the responses of these pixels have resolvable structure, indicating that there is biologically useful data present. The use of the constrained analysis thus provides more complete and more reliable analyses.

**Figure 7:**
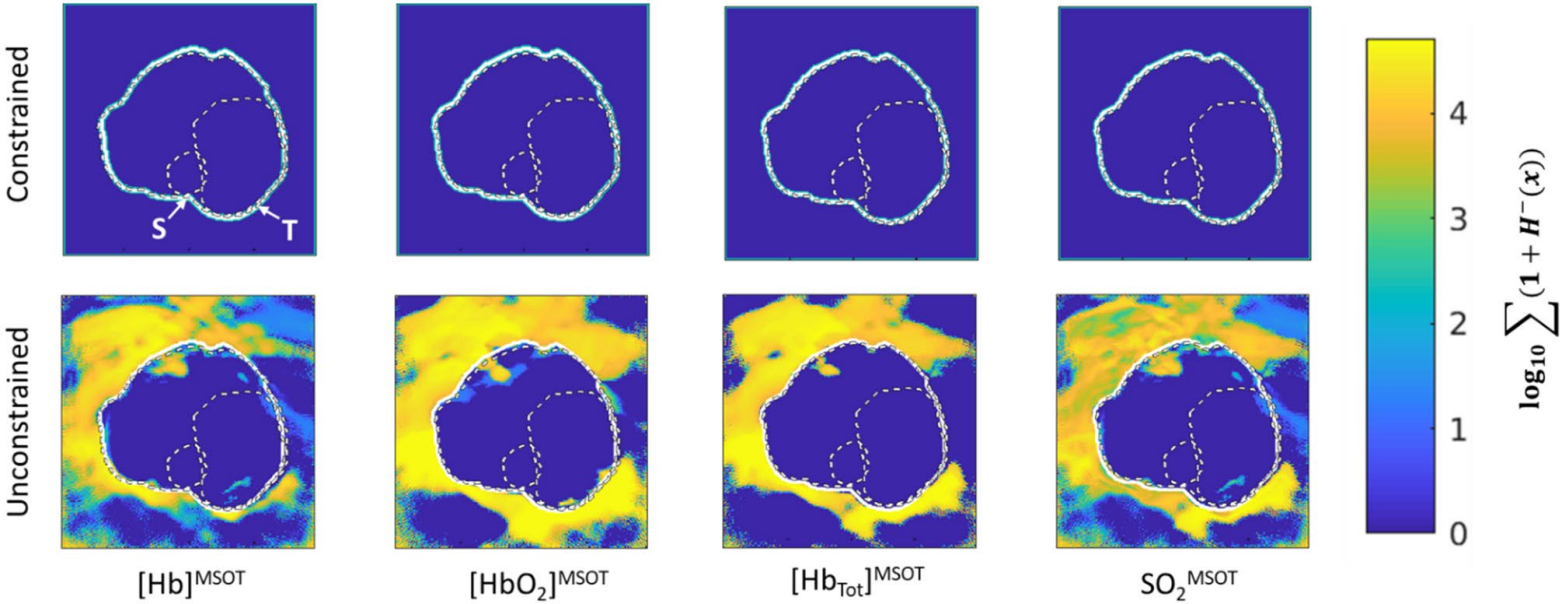
Total occurrence of negative pixels in each channel for the constrained and unconstrained analyses on Dataset A. Animal outline shown, S: Spine, T: Tumor. The constrained analysis results in no negative pixels at any point throughout the imaging time course, reflecting the consistent preservation of the non-negative constraint. The unconstrained analysis, by contrast, develops a large number of negative pixels, including numerous regions within the animal. Figure created using a combination of MATLAB and Microsoft PowerPoint.

**Figure 8:**
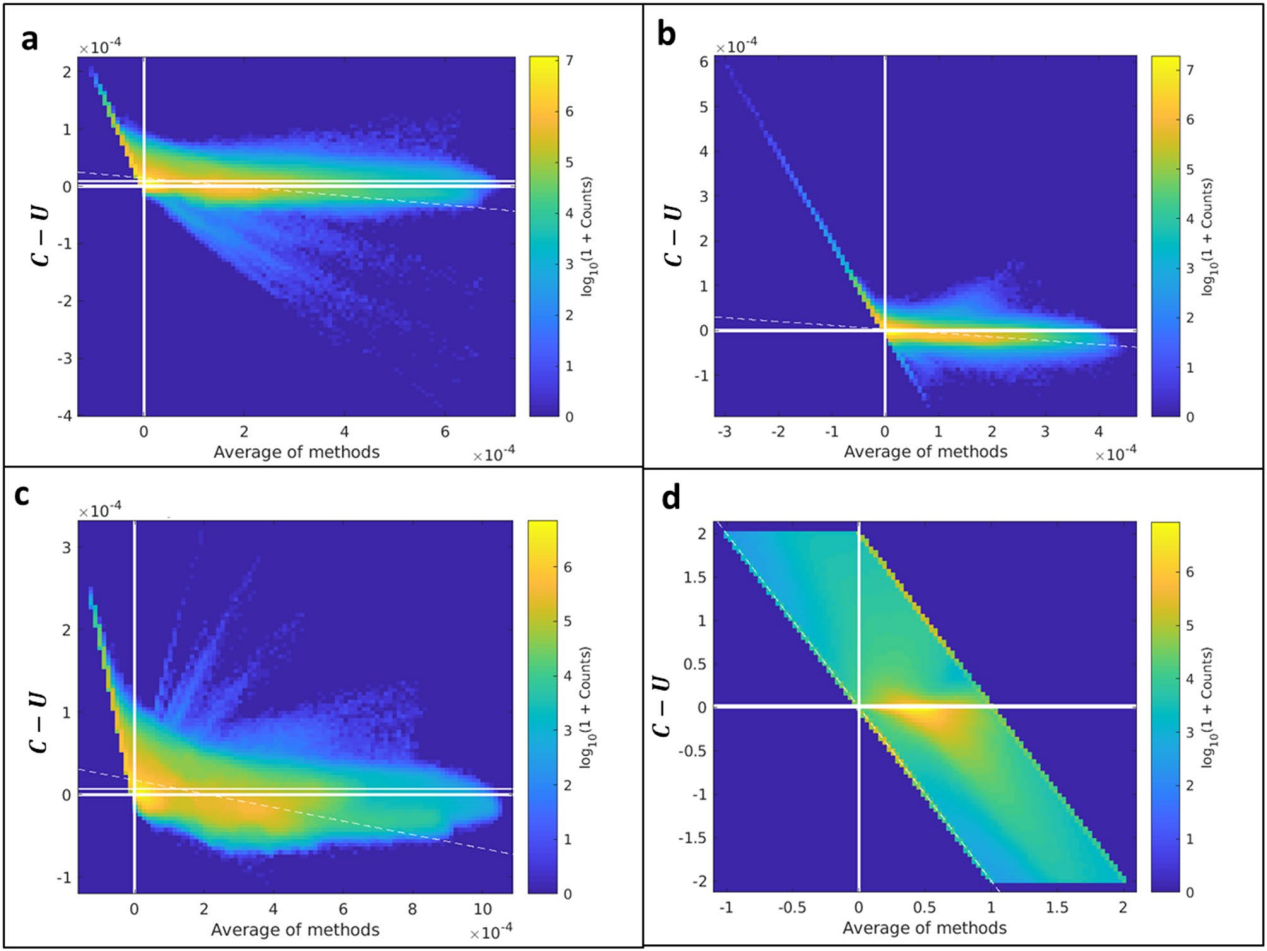
Bland–Altman plots between the constrained and unconstrained analyses on Dataset A. (**a**) [HbO_2_]^MSOT^, (**b**) [Hb]^MSOT^, (**c**) [Hb_tot_]^MSOT^, (**d**) SO_2_^MSOT^. There is generally good agreement throughout all channels, though there appears to be a consistently lower estimate of all parameters using the constrained approach. The appearance of diagonal structure in (**a**–**c**) is due to the non-negative constraint, while the band of finite width in (**d**) is the result of the constrained method’s SO_2_ estimates being confined to [0,1]. Figure created using a combination of MATLAB and Microsoft PowerPoint.

As applied to Dataset A, the effects of processing choice are salient: Fig. 7 shows the total number of negative-valued pixels throughout the study. The constrained analysis yields no negative values, while the unconstrained analysis results in negative values in various locations through various channels. Even within the tumor bulk, there are substantial regions where the values of [HbO_2_]^MSOT^, [Hb]^MSOT^, and SO_2_^MSOT^ attain negative values. The presence of these values would compromise biological inference due to the non-physicality of negative values. Overall differences between the methods for each channel are shown in the Bland–Altman plots (Fig. 8). There is generally good agreement between each, though the constrained method tends to provide lower estimates than the unconstrained method. The presence of an overall diagonal structure reflects the non-negativity of the constrained analysis.

**Figure 9:**
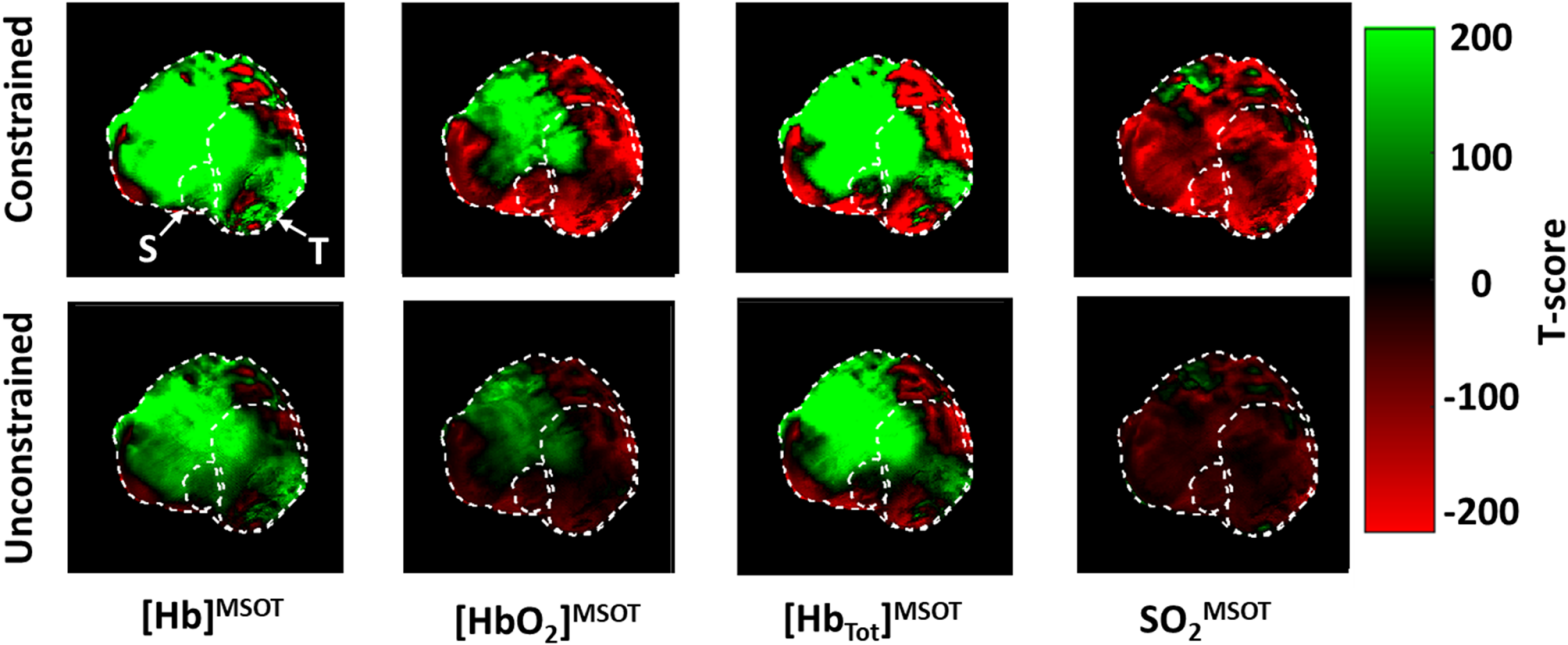
T-scores of difference in each parameter before and after CA4P administration in Dataset A. Across all channels, the constrained method results in T scores with substantially increased magnitude, corresponding to greater significance. The large positive region in the upper-left of the [Hb]^MSOT^_,_ [HbO_2_]^MSOT^, and [Hb_tot_]^MSOT^ is likely due to physiological effects unrelated to the local vascular effects of CA4P or the choice of processing. This effect is normalized in the ratiometric calculation of SO_2_^MSOT^. Figure created using a combination of MATLAB and Microsoft PowerPoint.

**Figure 10:**
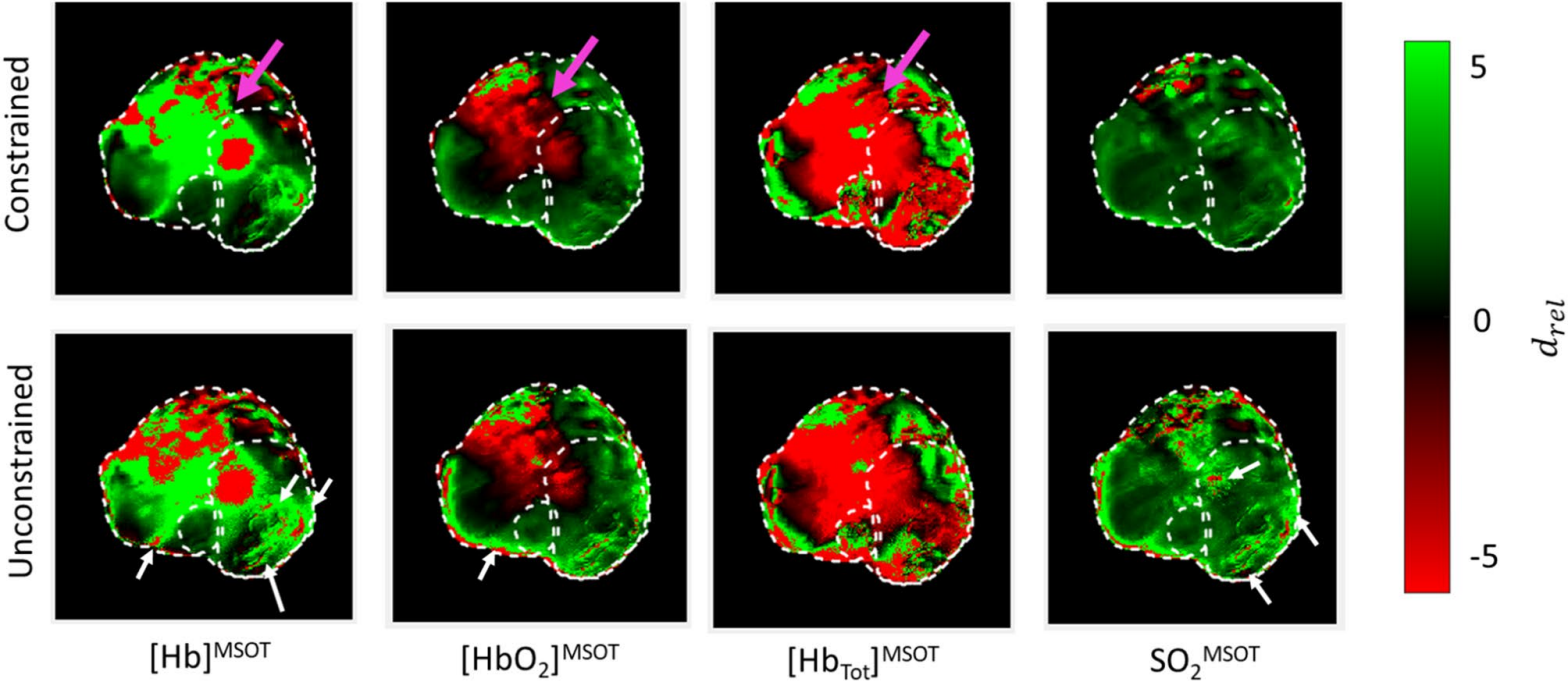
Relative Cohen’s d (Supplementary Information) of drug administration calibrated against oxygen-air transition during initial gas challenge in Dataset A. In both constrained and unconstrained [Hb]^MSOT^ and [HbO_2_]^MSOT^ images, there is a large region of anomalous relative effect (magenta arrows), likely due to changes in measured [Hb_tot_]^MSOT^ over the course of the experiment. This large region is absent in the SO_2_^MSOT^ analyses due to the ratiometric calculation. More localized artifacts such as those highlighted in the unconstrained analysis (white arrows) are due to modelling or reconstruction inaccuracies, and so are not compensated by calculating SO_2_^MSOT^. Figure created using a combination of MATLAB and Microsoft PowerPoint.

When examining changes in response to treatment, the constrained approach leads to greater significance when comparing each of the parameter values before and 60 min after administration of CA4P (Fig. 9), allowing the resolution of significant changes even in low-signal areas. Similar results were seen when considering the relative effect of the drug administration normalized by the oxygen-air transition response (Fig. 10). There is a large region of anomalous response in the [Hb]^MSOT^ and [HbO_2_]^MSOT^ channels, signified by the magenta arrows in Fig. 10, for both the constrained and unconstrained methods. These variations are attributable to variations in [Hb_tot_]^MSOT^ , which may themselves be due to temperature-dependent signal changes in the animal^54,55^ or systemic blood pressure effects due to CA4P administration^56^. As a result, SO_2_^MSOT^ provides a more reliable metric of variation due to its intrinsic calibration against [Hb_tot_]^MSOT^. Similarly, SO_2_^MSOT^ is less sensitive to variations in light fluence, likely due to variations in light spectrum as a function of wavelength being weaker throughout the bulk of the animal than the variations in light intensity. The unconstrained approach results in a greater number of small-scale anomalous responses (Fig. 10, white arrows) due to the non-physical values of various parameters in those pixels. In contrast, the relative effect of the constrained SO_2_^MSOT^ is much more consistent, showing vessel-like structures in the response within the tumor bulk, with a dramatically reduced occurrence of anomalous responses.

**Figure 11:**
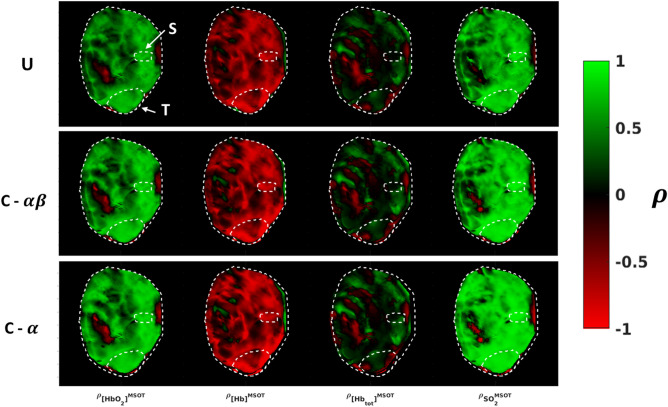
Correlation images against known inhaled O_2_ time course for Dataset B. U: Unconstrained analysis. C-$\alpha \beta$: Constrained analysis using $\alpha \beta$ Kalata filter. C-$\alpha$: Constrained analysis using $\alpha$ Kalata filter. The use of the constrained approach results in improved Pearson’s correlation ($\rho$) with the gas challenge pO_2_ time course across all channels. Figure created using a combination of MATLAB and Microsoft PowerPoint.

**Figure 12:**
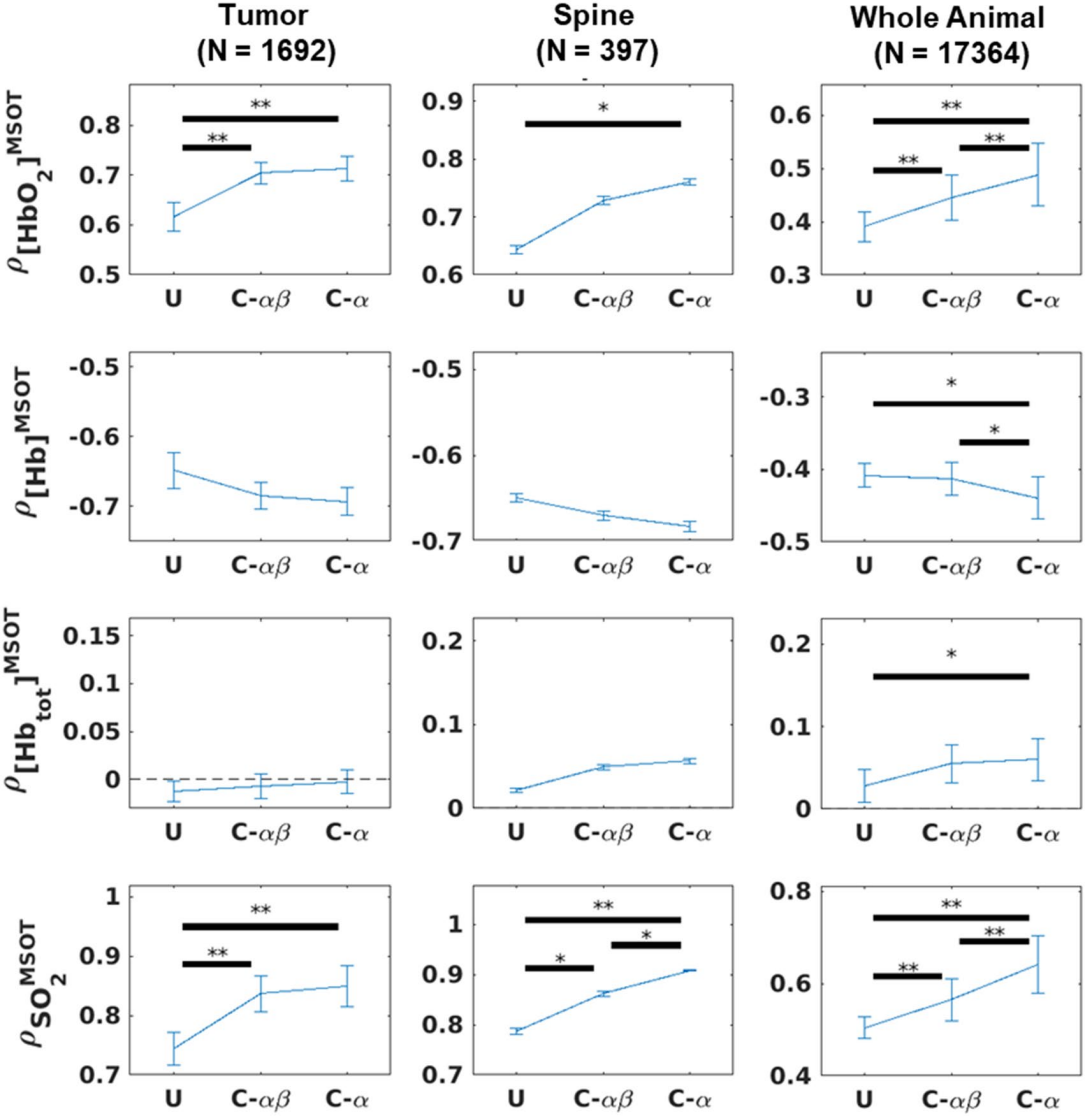
Plots of average correlation for distinct ROIs of Dataset B. A general improvement in correlation is seen using either of the constrained approaches, though the superior noise-rejection properties of the $\alpha$ filter enable it to achieve better overall correlation with the gas challenge pO_2_ time course. Figure created using a combination of MATLAB and Microsoft PowerPoint.

**Figure 13:**
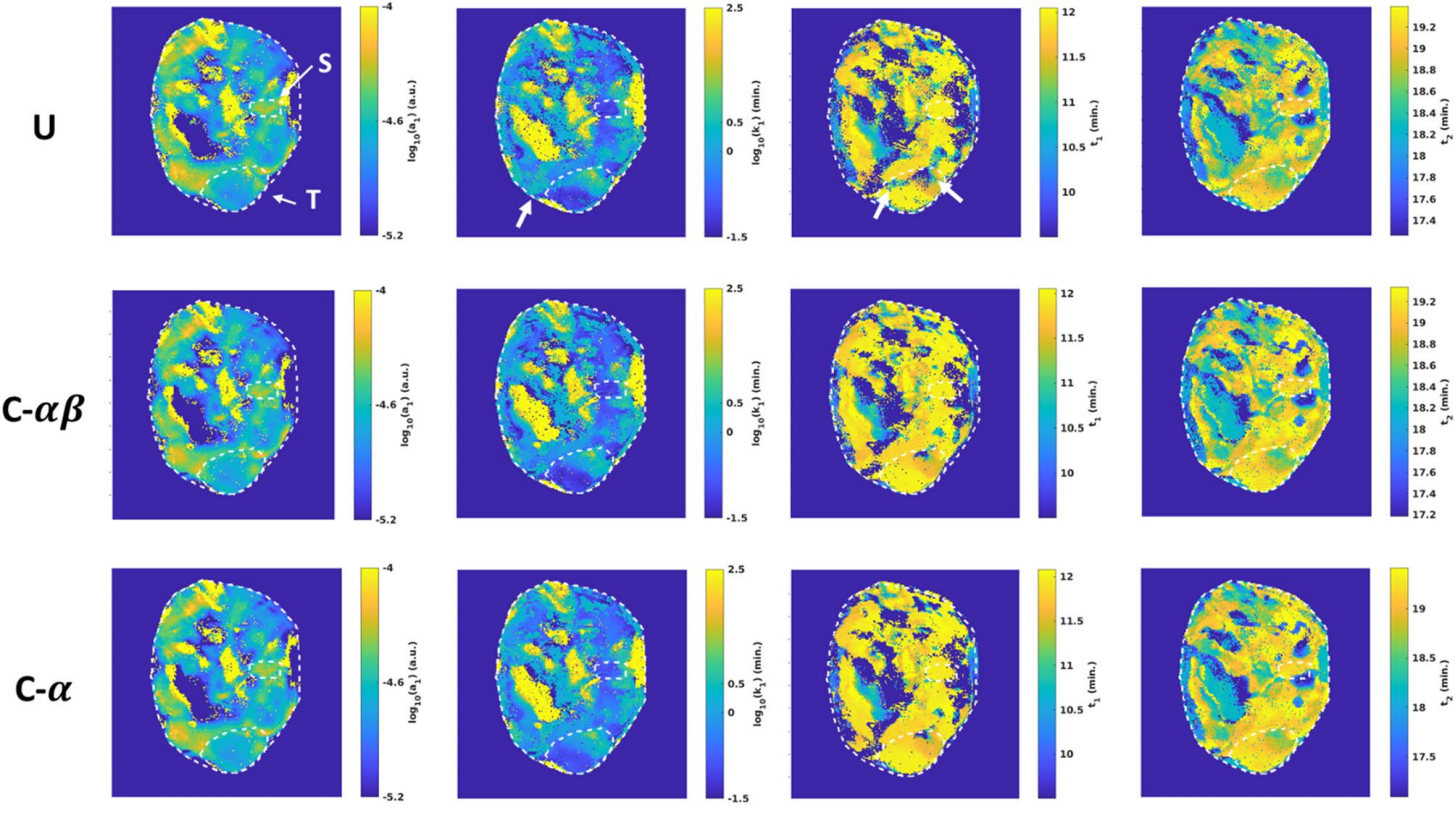
Selected parameters from fitting Dataset B using 7-parameter monoexponential model (Supplementary Information) using three different processing approaches. U: Unconstrained analysis. C-$\alpha \beta$: Constrained analysis using $\alpha \beta$ Kalata filter. C-$\alpha$: Constrained analysis using $\alpha$ Kalata filter. Although performance of the model fit is similar across much of the imaged area, the unconstrained analysis assigns inaccurate values of switching times to a large region on the interior of the tumor (white arrows). Figure created using a combination of MATLAB and Microsoft PowerPoint.

Increases in correlation against the known pO_2_ waveform were seen for both the $\alpha$ and $\alpha \beta$ constrained methods when compared to the unconstrained approach (Figs. 11, 12). Though the distributions of correlation were similar (Fig. 11), the constrained methods improved the positive correlation with [HbO_2_]^MSOT^ and SO_2_^MSOT^ and the negative correlation with [Hb]^MSOT^, while having a relatively mild effect on [Hb_Tot_]^MSOT^ (Fig. 12). Despite the visual similarities in correlation values between $\alpha$ and $\alpha \beta$ filtered timeseries (Fig. 11), the $\alpha \beta$ filter has the advantage of quickly following dynamic changes, while the $\alpha$ filter naturally suffers from a lag time after such changes. Nevertheless, the $\alpha$ filter suppresses noise effectively, leading to the improved correlations shown in Fig. 12. The improvements in correlation were particularly significant in the HbO_2_ and SO_2_ channels, possibly reflecting the suitability of these channels for measuring response to gas challenge. The constrained methods were also able to achieve better model fits, as seen in Fig. 13. The unconstrained approach provided results which were generally consistent with the constrained approaches, but resulted in many noncausal switching time values in the tumor region.

**Figure 14:**
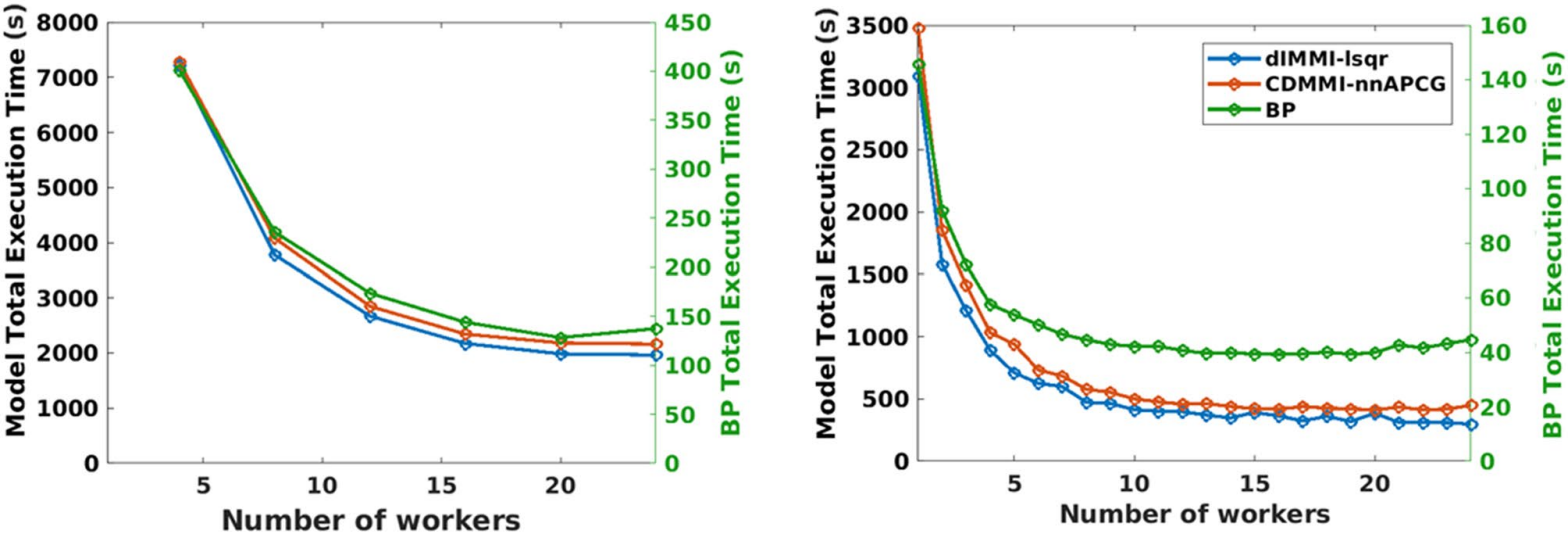
Reconstruction using different algorithms for varying numbers of parallel workers. Both Dataset A (left) and Dataset C (right) benefit from parallelization, though both show diminishing returns. When reconstruction time is normalized to the time required for a single worker (left) or 4 workers (right) to execute, it becomes clear that both model-based approaches benefit greatly from parallelization, while backprojection (BP) more rapidly saturates due to the greater proportion of its reconstruction which is non-parallelizable. For large numbers of workers, it becomes evident that system overhead becomes restrictive, as there is a decrease in relative speedup. Note separate axes for dIMMI and CDMMI (left) versus BP (right). Figure created using a combination of MATLAB and Microsoft PowerPoint.

The process of reconstruction benefits from parallelization, enabling faster processing. As shown in Fig. 14, even a few additional logical cores, as is available on most modern processors, provided dramatically improved performance. The model-based approaches generally benefited more from this parallelization; due to the larger proportion of the processing time per-frame taken up by the solution process itself, increased parallelization provides greater benefit to the model-based reconstructions. Diminishing returns with increasing numbers of processors are attributable to network and hard-disk limitations. We note that the scaling performance is comparable for each method, indicating that the relative overhead of parallelization for each method is comparable.

## Discussion

The MSOT-RAFT provides a common platform for reconstructing optoacoustic tomography data in a variety of scenarios. The package is open-source and may be scaled to accommodate the computing resources available. Configuration of the overall pipeline during preprocessing enables the straightforward usage of default processing approaches, while simultaneously providing the flexibility for more sophisticated modification of the processing path. We note that this publication is a static record, and recommend examining the Zenodo repository for any updates.

The MSOT-RAFT is accessible through a variety of interfaces, whether as a MATLAB toolbox or as a compiled executable library usable through other methods, with imminent deployment of Docker images capable of running on Singularity-enabled high performance computing environments. This allows it to be flexibly used in a broad array of contexts, whether running on an individual machine or as a component of a much larger distributed processing pipeline. There are numerous additional factors to consider; light fluence $\phi \left( {\vec{r},\vec{\lambda }} \right)$ is inhomogeneous throughout the imaging region, owing to the removal of photons via absorption by superficial layers. Fluence is also inhomogeneous through wavelength, due to the generally inhomogeneous distribution of endmembers within superficial layers. These additional considerations necessitate additional processing steps, which could be conveniently added through the modular structure of the framework.

The end goal of imaging is the ability to quantitatively resolve the spatial distribution of a variety of parameters, and the reconstruction procedures presented may be augmented for truly quantitative measurements. The reconstructed photoacoustic image is an inversion of a linear map representing the time-distance relationship under the assumption of zero acoustic attenuation and homogenous speed of sound. As others have noted, improved image quality can be attained with spatially-variant time-distance relationships^57,58^. The determination of the local speed of sound could be performed through Bayesian-type methods^58^, geometric simplification, or adjunct imaging such as transmission-reflection ultrasound^59^.

Though the MSOT-RAFT is presently developed for the input of photoacoustic imaging data acquired using the iThera MSOT imaging systems, we note that this is a question of input format; additional manufacturers and even experimental systems may generate data, which could be successfully reconstructed after suitable input formatting. We anticipate that the MSOT-RAFT will provide an effective starting point for future developments. In particular, the modular structure of the pipeline, and the end-to-end comparisons of reconstruction quality, enable external optimization of the entire system, so as to achieve optimal performance. Since the RAFT is parameter-driven, it could be tuned using a hyperparameter optimizer such as Spearmint in order to create more effective reconstruction pipelines^60–62^. The resulting optimized pipeline could then be recorded and shared, enabling more rapid dissemination of successful processing motifs.

The RAFT is broadly broken down into modular steps, and they do not all need to be performed using the toolbox itself. Indeed, one could use the RAFT for a large portion of the analysis, and inject additional processing to the analytical chain. This provides for extensive future developments, for example adding fluence correction prior to spectral unmixing.

We plan to extend the filter interface to allow for the calling of external subroutines, such as efficient GPU kernels, compiled C and C++ functions, and various Python scripts. We additionally hope to add support for standard imaging formats such as DICOM or OME-XML, to enable management of data using PACS systems and inclusion in other studies and databases ranging from the experimental to the clinical.

## Conclusion

We have described the implementation and demonstrated the performance of MSOT-RAFT, an open-source toolbox for processing and reconstructing photoacoustic imaging data, and have demonstrated its use for processing and analyzing photoacoustic imaging data.

## Supplementary Information

Supplementary Information.

## Data Availability

The most up to date publicly available version of the RAFT, along with examples of usage and test data, can be found at https://doi.org/10.5281/zenodo.4658279 and is available under the MIT license.

## Data availability

All data analyzed in this study are available from the corresponding author (RPM) on reasonable request.
