# Supplementary material for: A scalable open-source MATLAB toolbox for reconstruction and analysis of multispectral optoacoustic tomography data
Source: Sci Rep. 2021 Oct 6;11:19872. doi: 10.1038/s41598-021-97726-1 (PMC8494751; doi:10.1038/s41598-021-97726-1)
Supplement: Supplementary file 1 — Supplementary Information. [file 41598_2021_97726_MOESM1_ESM.docx]

# Supplementary Information

## Calculation of relative effect

Calculation of the relative effect of an intervention was defined as the ratio of Cohen’s *d* values of a test response against a reference response.

Cohen’s D is calculated as:

|  | $d=\frac{\bar{x}_{2}-\bar{x}_{1}}{s}$ | (S1) |
| --- | --- | --- |

where $\bar{x}_{2}$ and $\bar{x}_{1}$ refer to the mean value of a parameter before ($x_{1}$) and after ($x_{2}$) an experimental intervention, while $s$ denotes the pooled standard deviation of the parameter, given by

|  | $s=\sqrt{\frac{\left( n_{1}-1 \right)s_{1}^{2}+\left( n_{2}-1 \right)s_{2}^{2}}{n_{1}+n_{2}-2}}$ | (S2) |
| --- | --- | --- |

where $s_{1}$ and $s_{2}$ refer to the standard deviation of $x_{1}$ and $x_{2}$, respectively, and $n_{1}$, $n_{2}$ refer to the number of samples of each parameter.

The relative effect size is then given by

|  | $d_{rel}=\frac{d_{test}}{d_{ref}}$ | (S3) |
| --- | --- | --- |

In the case of Dataset A, we used the internal gas challenge as a reference against which the effects of the CA4P intervention were compared, such that

|  | $d_{ref}=\frac{\bar{x}_{ox,1}-\bar{x}_{air,2}}{s}$ | (S4) |
| --- | --- | --- |

Where $\bar{x}_{ox,1}$ refers to the mean value during the first oxygen-breathing component of the gas challenge after the first gas transition, and $\bar{x}_{air,2}$ refers to the mean value during the second air-breathing component of the gas challenge, after the second gas transition.

|  | $d_{test}=\frac{\bar{x}_{ox,2,pre}-\bar{x}_{ox,2,post}}{s}$ | (S5) |
| --- | --- | --- |

Where $\bar{x}_{ox,2,pre}$ refers to the mean value during the second oxygen-breathing component of the gas challenge after the third gas transition but before the administration of CA4P, and $\bar{x}_{ox,2,post}$ refers to the mean value during the second air-breathing component of the gas challenge, at the end of the scan.

## Gas Challenge Model

The following piecewise function was used to model the [HbO_2_]^MSOT^ response to the gas challenge:

|  | $\left[ HbO_{2} \right]^{MSOT}\left( t \right)=\left\{ \begin{aligned} c_{o}+mt if t<t_{1} \\ c_{o}+mt+a_{1}\left( 1-\exp\left( -\frac{t-t_{1}}{k_{1}} \right) \right) if t\geq t_{1} and t\leq t_{2} \\ c_{o}+mt+ \left( 1-\exp\left( -\frac{t_{2}-t_{1}}{k_{1}} \right) \right)\exp\left( -\left( \frac{t-t_{2}}{k_{2}} \right) \right) if t>t_{2} \end{aligned} \right.$ | (S7) |
| --- | --- | --- |

## Supplementary Figures


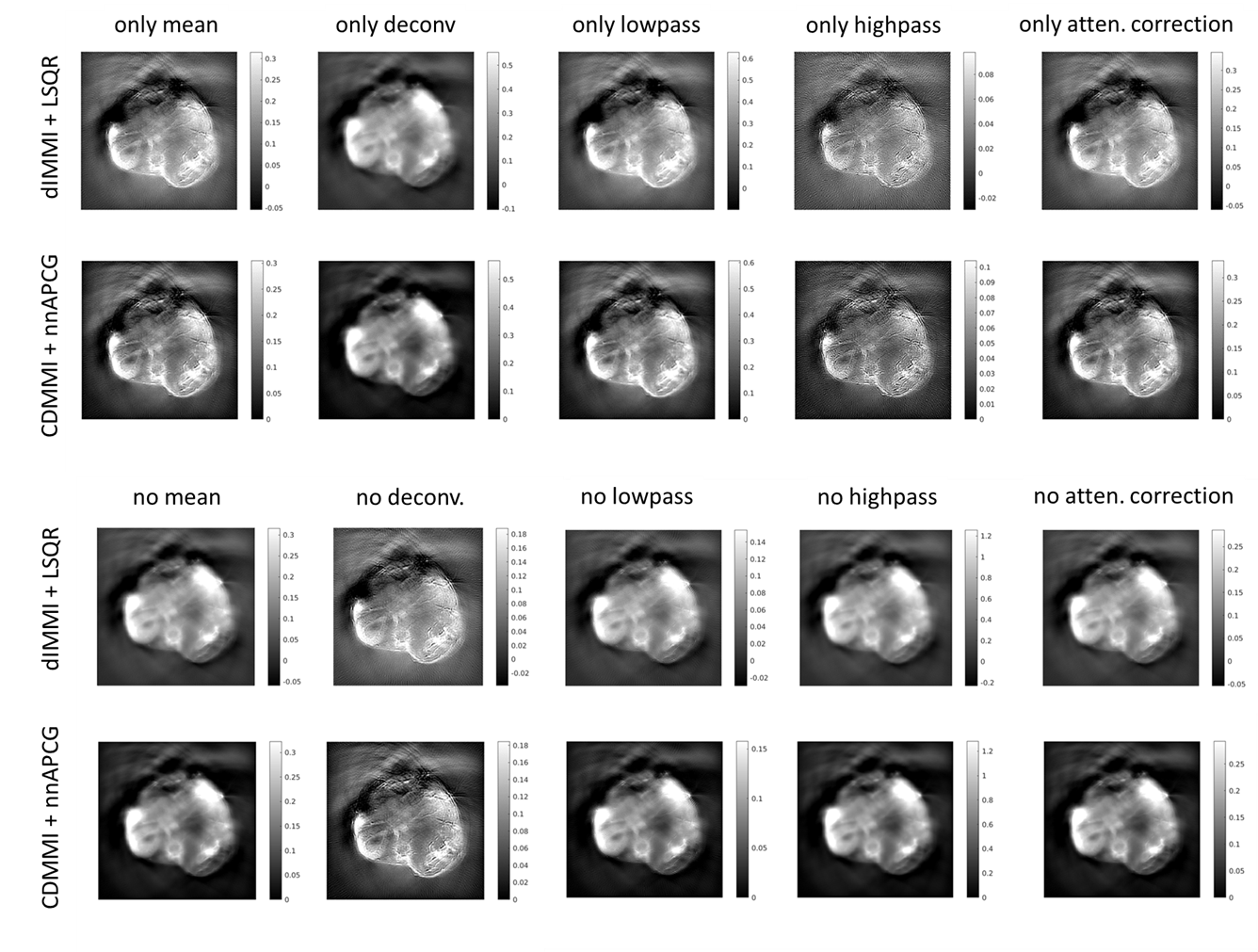


**Supplementary Figure 1: Reconstructions using leave-one-in and leave-one-out for each individual component of the overall prefilter.** Mean removal has a subtle effect on the overall reconstruction, as the offset is corrected on a per-transducer basis. This has the net effect of stabilizing the reconstruction process. Deconvolution addresses oscillatory artifacts around high-intensity regions, with the net effect of visually smoothing the image. The lowpass filter has a substantial effect on the absolute signal intensity, while the highpass filter alone results in high-frequency artifacts. Attenuation correction changes the overall scale of the image on a wavelength-dependent basis, and so is more relevant for downstream multispectral processing.
